# ELIXIR-UK role in bioinformatics training at the national level and across ELIXIR

**DOI:** 10.12688/f1000research.11837.1

**Published:** 2017-06-21

**Authors:** L. Larcombe, R. Hendricusdottir, T.K. Attwood, F. Bacall, N. Beard, L.J. Bellis, W.B. Dunn, J.M. Hancock, A. Nenadic, C. Orengo, B. Overduin, S-A Sansone, M. Thurston, M.R. Viant, C.L. Winder, C.A. Goble, C.P. Ponting, G. Rustici

**Affiliations:** 1 MRC Human Genetics Unit, The Institute of Genetics and Molecular Medicine, University of Edinburgh, Edinburgh, EH4 2XU, UK; 2 School of Computer Science, University of Manchester, Manchester, M13 9PL, UK; 3 Department of Genetics, University of Cambridge, Cambridge, CB2 3EH, UK; 4 Birmingham Metabolomics Training Centre, School of Biosciences, University of Birmingham, Birmingham, B15 2TT, UK; 5 Earlham Institute, Norwich, NR4 7UZ, UK; 6 The Software Sustainability Institute, University of Manchester, Manchester, M13 9PL, UK; 7 University College London, London, WC1E 6BT, UK; 8 Edinburgh Genomics, University of Edinburgh, Edinburgh, EH9 3FL, UK; 9 Oxford e-Research Centre, University of Oxford, Oxford, OX1 3QG, UK

**Keywords:** ELIXIR-UK, Training, Bioinformatics, Genomics, Metabolomics, Structural Bioinformatics, Impact, TeSS

## Abstract

ELIXIR-UK is the UK node of ELIXIR, the European infrastructure for life science data. Since its foundation in 2014, ELIXIR-UK has played a leading role in training both within the UK and in the ELIXIR Training Platform, which coordinates and delivers training across all ELIXIR members. ELIXIR-UK contributes to the Training Platform’s coordination and supports the development of training to address key skill gaps amongst UK scientists. As part of this work it acts as a conduit for nationally-important bioinformatics training resources to promote their activities to the ELIXIR community. ELIXIR-UK also leads ELIXIR’s flagship Training Portal, TeSS, which collects information about a diverse range of training and makes it easily accessible to the community. ELIXIR-UK also works with others to provide key digital skills training, partnering with the Software Sustainability Institute to provide Software Carpentry training to the ELIXIR community and to establish the Data Carpentry initiative, and taking a lead role amongst national stakeholders to deliver the StaTS project – a coordinated effort to drive engagement with training in statistics.

## Introduction

ELIXIR-UK is at the forefront of bioinformatics training provision, across the UK and in the wider ELIXIR training programme. At the national level, ELIXIR-UK has surveyed bioinformatics training needs, has identified training gaps and developed strategies to address them. This resulted in the establishment of successful national training initiatives (see “ELIXIR UK training in the UK” section), focusing on up-skilling the UK research community, and developing a coherent bioinformatics training programme across the UK, coordinating the activities of a growing network of bioinformatics training centres. Three of these centres have also been recognised as ELIXIR-UK resources, through an open and transparent mechanism for identifying resources suitable for contribution to ELIXIR ^1^: these are the Bioinformatics Training programme of the University of Cambridge; the Birmingham Metabolomics Training Centre; and the Edinburgh Genomics training programme. Together, these centres offer in excess of 150 high-quality practical bioinformatics training courses each year, focusing on a wide range of topics, from basic bioinformatics skills to advanced data analysis; over 2,500 people (largely UK-based scientists) are trained every year by these three centres combined. Alongside ELIXIR’s flagship Training Portal, TeSS, which is also developed and run by ELIXIR-UK, these three training centres are a major contribution from the Node to the ELIXIR-wide training programme, and are committed to adhere to the ELIXIR’s standards for quality, discoverability and interoperability.

Additionally, ELIXIR-UK has played an ELIXIR-wide strategic role in training by co-leading and coordinating, together with ELIXIR-NL and ELIXIR-CH, the ELIXIR Training Platform [Prof Ponting (Lead) and Dr Hendricusdottir (Coordinator), from 2014 to 2017; and currently Dr Bellis (Coordinator)], which aims to build a sustainable training infrastructure across Europe, and deliver training based on the evolving needs and requirements of the scientific community (See “ELIXIR-UK’s role in the ELIXIR Training Platform” section). ELIXIR-UK has also been involved in establishing and chairing the ELIXIR Training Coordinators Group (TrCG, Dr Hendricusdottir). The TrCG comprises training representatives from each of the ELIXIR Nodes, and plays an important role in coordinating ELIXIR-wide training efforts, leading the implementation of ELIXIR’s training strategy across Europe. This report provides an overview of ELIXIR-UK’s role in bioinformatics training at the national level and across ELIXIR.

## ELIXIR-UK training in the UK

*Please note that the activities listed in this section have been mapped to the original timetable of activities from the BB/L005069/1 grant ( Supplementary File 1).*

At the start of the ELIXIR-UK activities, a scoping exercise was undertaken (June to October 2014, Dr Larcombe), which involved working with UK sector leads to consider the training needs of their communities, and carrying out an industry survey of skills needs ( *Supplementary File 1* | Year 1 - T2, T3). The result of this activity was the identification of 5 priority areas ( *Supplementary File 1* | Year 1 - T2, T3, T5): Core Bioinformatics Skills, Metabolomics, Structural Bioinformatics, Clinical Genomics, and Applied Genomics. The rationale for prioritising these 5 thematic areas came from community input and training-gap analysis. Additionally, the emergence of Core Bioinformatics Skills, representing a broad base of quantitative and computational skills, also arose from responses to the ELIXIR-UK industry survey, and trends emerging from the BBSRC/MRC vulnerable skills report and the Global Organisation for Bioinformatics Learning, Education & Training (GOBLET) survey.

The complete results of the industry survey are available from the ELIXIR-UK website, but some key indicators are presented here:

- Around **70%** of bioinformaticians would like training in statistics and data-analysis methods, with a specific focus on sequencing and genomics.
- Similarly, around **60%** of wet-lab biologists would like to acquire skills in data visualisation, data manipulation and general statistics.
- Interestingly, the skills desired by wet-lab scientists are also those that bioinformaticians ( **70–80%**) consider to be the most crucial competencies.
- The majority of wet-lab scientists ( **74%**) have no programming experience, and **60%** perform their data analysis in Excel.
- When considering the use of statistical methods, **65%** are not confident with statistics, with a small percentage of respondents not even sure what statistical knowledge they should have ( **6%**).
- Although most respondents do have a bioinformatician with whom they can collaborate, **34%** do not have a bioinformatician/statistician to whom they can turn for support.

ELIXIR-UK has been addressing the 5 priority training areas listed above through:

- Coordinating a combination of named ELIXIR-UK training centres;
- Developing the ELIXIR training portal, TeSS, which provides a snapshot of ELIXIR’s training landscape by making training (events, materials, *etc*.) discoverable and accessible;
- Collaborating with other institutes and training initiatives;
- Working with other infrastructure projects; and
- Developing new projects to fill existing training gaps.
- How this has developed is outlined in the following sections for each of the 5 priority areas.

### Core Bioinformatics Skills ( Supplementary File 1 | Year 2 - T2, T3, T5)

This area of the ELIXIR-UK training plan closely aligns with skills needs established by the BBSRC/MRC vulnerable skills report, and the skills gaps observed across ELIXIR and beyond. Increasing training in fundamental computational and quantitative skills is an area in which ELIXIR-UK has been very successful, with established partnerships with both Software Carpentry (SWC) and Data Carpentry (DC), and taking a leading role in the development of a national training strategy in statistics.

***SWC/DC training.*** Through its partnership with the Software Sustainability Institute (SSI), **ELIXIR-UK has initiated and coordinated the provision of SWC/DC training across ELIXIR**. These courses teach the fundamental skills necessary for data manipulation and best practices for reproducible research. Over the past 3 years, more than 50 workshops have been organised in the UK, aiming to teach researchers with minimal or no computational skills some basic data-manipulation and software-development techniques to help them improve or speed up their research. Feedback from course participants is extremely positive, and the topics covered by SWC/DC training are considered fundamental for all researchers. **ELIXIR-UK has had a pivotal role** in establishing SWC/DC training programmes within ELIXIR during the 2015 Pilot project, and helping to build the capacity within Nodes for sustaining these programmes independently. In 2016, ELIXIR began drafting a collaboration agreement with the SWC/DC Foundations, which will cover the development of common training materials, the coordination of training workshops for researchers, and the construction of a community of certified ELIXIR trainers. ELIXIR-UK will continue to provide support to such coordination effort, and the SSI is now working on establishing a SSI in Europe.

In the future, ELIXIR-UK wishes to expand the delivery of SWC/DC courses across the UK, by organising courses in locations where these have not been run before, to increase training capability in the UK. The collaboration agreement between ELIXIR and the SWC/DC Foundations will only allow for the delivery of two workshops per year at each ELIXIR Node, which would not be sufficient to meet the UK Institutions’ demand for such training. Some of the remaining funds from the original ELIXIR-UK BBSRC grant (BB/L005069/1) will enable this, while additional funding opportunities are being explored to continue sustaining this training provision in the long term.

***Statistics training.*** Over the last few years, several professional bodies, societies, industry bodies and the Research Councils have surveyed skills requirements, with statistics consistently being highlighted as an acute need. Specifically, from the ELIXIR-UK skills survey distributed to industry wet-lab biologists, 63% of respondents were not confident in their use of statistics. Because of this trend, ELIXIR-UK contributed to the development of the Statistics Training Signposting project, and has taken the lead in promoting statistics skill schools, helping researchers to: (i) realise that statistics is something that everyone should be able to do, and (ii) acquire a basic skill-set to enable them to learn “practical” statistics. BBSRC is currently funding the development of statistics skill schools, in partnership with ELIXIR-UK, and the dissemination of training materials through online resources. This activity is led by the University of Cambridge (Dr Rustici). The first course from this project was run in 2016, with financial support from Cancer Research UK, and resulted in extremely positive feedback. Additional courses will run in Cambridge between 2017 and 2019, with support from a BBSRC STARS award (Dr Rustici). Course materials will be disseminated online, made discoverable through TeSS, and linked to follow-up training; this project also relies on additional financial contribution from the MRC, plus training materials from AstraZeneca. Statistics courses will also be added to the DC training provision, to link these initiatives and increase sustainability.

The University of Oxford (Dr Sansone) will contribute to statistics training with the community-driven STATistics Ontology (STATO), which is being developed also as a didactic tool. STATO aims to cover processes such as statistical tests, their conditions of application, and information needed or resulting from statistical methods, such as probability distributions, variables, spread and variation metrics. STATO also covers aspects of experimental design, and description of plots and graphical representations commonly used to provide visual cues of data distribution or layout, and to assist the review of results. Funds will be sought to enhance the didactic aspect of STATO under the UK Node activities.

Statistics has also been identified as an area of need in other ELIXIR Nodes, so the statistics training developed by ELIXIR-UK will also contribute to and inform the wider ELIXIR training activities in this area.

### Metabolomics ( Supplementary File 1 | Year 2 - T2, T3, T5)

Metabolomics was quickly identified as an area of growing need: ELIXIR-UK (University of Birmingham: Prof Viant, Dr Dunn, Dr Weber and Dr Winder) carried out a metabolomics community survey to assess resource usage, researcher base, and skills needs in partnership with the international Metabolomics Society. This activity highlighted several important community needs, and specifically demonstrated the sector's rapid growth, and the resulting large number of researchers new to this subject, who therefore need training. A review of this survey is published ^2^, and a summary of all responses is available online.

An early reaction to this was to facilitate funding and knowledge exchange (Dr Larcombe) between ELIXIR-UK partners (QMUL, Prof Bessant; and Birmingham) to maximise the impact of BBSRC MTP funds for developing proteomics and metabolomics bioinformatics training, with resulting courses being developed collaboratively. To extend its reach, the metabolomics bioinformatics course is being operated online as a Small Private Online Course ( SPOC). The course was first run in February 2017, and 51 trainees completed the course from the UK, Europe and Asia. Additionally, observations from this survey formed part of the rationale for the proposal and development of the Birmingham Metabolomics Training Centre, alongside the existing operation of two UK metabolomics cores: the Phenome Centre in Birmingham (through a £5M MRC-funded grant) and the NERC Metabolomics facility (NBAF-B). This was a significant development, and the Birmingham Metabolomics Training Centre is now a named ELIXIR-UK resource, offering several annual face-to-face, hands-on courses, a well-subscribed MOOC, through FutureLearn, and a SPOC focused on computational processing and analysis of metabolomics data. Industry (Thermo Fisher Scientific and Waters Ltd.) has recognised the importance of this training centre, and has provided funds (£1M) for scientific instrumentation and software to enhance training activities and capabilities for the trainees.

The centre has taken an important lead in the development of the European metabolomics training initiative (EmTraG), which was formed in 2016 with the support of ELIXIR-UK. EmTraG was created to address a pressing need to harmonise the rapidly expanding portfolio of metabolomics training courses in Europe, and improve its scientific coverage, geographical reach, quality and impact; ultimately, to empower the next generation of analytical, computational and applied metabolomics scientists. EmTraG seeks close ties with the ELIXIR Training Platform at a European level, and the Metabolomics Society at an international level. It is intended that www.EmTraG.eu will serve as the principal European portal for metabolomics training with close links to other portals, including TeSS.

Whether ELIXIR-UK will continue to play a role in delivering further skills training in this area is unclear, given the resources currently in place in Birmingham. The UK Node will continue to seek funding in order to support the Birmingham Training Centre as a Node resource, and play a key advocacy role to provide routes to training at the European level. Some of the remaining funds from the original ELIXIR-UK BBSRC grant (BB/L005069/1) will be used to: (i) fund bursaries to enable BBSRC and MRC PhD students to attend face-to-face training courses, covering all of the course fee, and (ii) continue to operate an online course focused on metabolomics data processing and statistical analysis.

### Structural bioinformatics ( Supplementary File 1 | Year 2 - T2, T3, T5)

The field of structural bioinformatics, including both macromolecular structure and small chemical structure-related bioinformatics, is critical to translational medicine and the development of new therapeutics/medicines. The UK has a strong and historic community in this field, covering a great deal of expertise. Correspondingly, there are some key tools that have been developed, and for which the availability of training would be beneficial.

The early stages of ELIXIR-UK activity in this sector brought together members of this community to define the scope of training need, and to discuss training approaches for tackling complex concepts and methodologies (UCL, Prof Orengo). This process identified workflow-based approaches as an ideal way to present complex procedural tasks in structural bioinformatics, both as a high-level depiction of methodologies, and as a signpost to available training materials for distinct experimental/software stages. Unfortunately, the lack of funding made it impossible to implement these training workflows or develop face-to-face courses, with the exception of a small number of training activities (two events per year) in the analysis of protein structure data, which are run at UCL/EMBL-EBI and University of Cambridge.

The promotion of workflows as a high-level training “map” has nevertheless influenced the development of the ELIXIR-UK training portal, TeSS (see below); however, additional funding is needed to consolidate these activities and implement structural bioinformatics workflows in TeSS. Some of the remaining funds from the original ELIXIR-UK BBSRC grant (BB/L005069/1) will be used to implement these workflows, to provide training in the use of 3D structures to predict the impacts of genetic variations.

### Clinical Genomics ( Supplementary File 1 | Year 2 - T2, T3, T5)

In 2014, a need for training in the field of clinical genomics became clear, given the aspirations of various groups to further the implementation of genomic medicine at a large scale. Principal amongst those groups was Genomics England, as it rapidly moved towards the start of the UK national 100,000 genomes project. As such, ELIXIR-UK felt that the development of training in this area was of critical importance to facilitate the clinical use, and interpretation, of genomics data.

Several universities across the UK are playing a strategic role in developing Clinical Bioinformatics training, particularly through collaboration with Health Education England (HEE) and Genomics England, focusing on training the healthcare workforce to make use of genomic data for patient care. Several UK Universities have recently set up MSc courses in Genomics Medicine, and have developed online learning resources in Clinical Bioinformatics, such as the MOOC from the University of Manchester. In addition, HEE is improving their Scientific Training (STP) and Higher Specialist Scientific Training (HSST) programmes in clinical bioinformatics and clinical genomics by incorporating SWC/DC courses as part of the official curriculum, to ensure scientists have the right training in data management and analysis, and computational skills.

It is unclear how these MSc programmes will continue to be funded once the initial HEE sponsorship finishes, but discussions are taking place to ensure that clinical bioinformatics training provision continues in the long term. ELIXIR-UK (Dr Rustici) is working in partnership with HEE to establish best practices in clinical bioinformatics training, and to delineate a core clinical bioinformatics curriculum.

### Applied Genomics ( Supplementary File 1 | Year 3 - T2, T3, T5)

Two training centres (University of Cambridge and Edinburgh Genomics) heavily contribute to the provision of training in applied genomics. Courses on the analysis of High-Throughput Sequencing (HTS) data represent the largest subgroup of training events run every year at these two sites, and cover a wide spectrum of sequencing application (including whole genome sequencing, RNA-seq, ChIP-seq, methylation, DNA-seq, single cell RNA-seq, small-RNA-seq, *etc*.). The commitment from both centres to continue providing training in this area remains very high.

In this context, ELIXIR-UK has been collaborating with the Global Organisation for Bioinformatics Learning, Education & Training (GOBLET) ^3^ on developing guidelines to enable training material sharing, dissemination and reuse. Common standards for describing training materials have been proposed and applied to the curation of existing HTS training materials ^4^, during two face-to-face meetings of trainers from the HTS and metagenomics communities. The first such workshops was sponsored by ELIXIR-UK. Additional curation events are planned in the near future; see the ‘External liaisons’ section for more details.

## ELIXIR-UK’s role in the ELIXIR Training Platform

ELIXIR-UK is currently responsible for three core activities within the ELIXIR Training Platform: (i) development of measures of training impact and quality assessment, (ii) development of the Training portal, TeSS, and (iii) coordination of the ELIXIR Training Platform. These activities are dependent on ELIXIR-EXCELERATE funding, which will terminate in 2019. Between 2014 and 2017, the Training Platform leadership and coordination (Prof Ponting and Dr Hendricusdottir) were funded by both ELIXIR-UK and ELIXIR-EXCELERATE.

### Training impact and quality assessment

The **training impact and quality assessment** work aims to **develop a framework for capturing and reporting on the impact of the ELIXIR training programme as a whole**, and to demonstrate the value gained from the time and money invested in training initiatives. As part of this work, a core set of Key Performance Indicators (KPIs) has been identified, and all ELIXIR training providers are in the process of implementing mechanisms (primarily through short- and long-term surveys) for collecting these KPIs for the training events taking place in their Nodes. Since February 2017, data from over 40 training events has been captured, with contributions from nine ELIXIR Nodes. This process will be iterated until an appropriate set of KPIs is identified that allows us to “measure” how participating in an ELIXIR training event has influenced how trainees work. This activity is co-led by the University of Cambridge (Dr Rustici, Dr Bellis) and EMBL-EBI (Dr Morgan). Collection of data through short-term surveys is being complemented with data collected through face-to-face interviews with course participants; these will be used to capture qualitative information, such as the improvement of understanding of a topic, or a subsequent change in professional development. Selected participants will be interviewed at the time of the event, and then at a defined point in the future, normally after 6 months to 1 year. Some of the remaining funds from the original ELIXIR-UK BBSRC grant (BB/L005069/1) will be used to run interviews in Cambridge, Birmingham, Edinburgh and at another ELIXIR Node.

Impact assessment is currently a high-priority within ELIXIR, not just for training, but for all of its services and resources; the approach being developed for measuring the impact of training events is now being used to assess the impact of other event types, such as BYOD workshops and Industry events. It is unclear how impact assessment will be supported after the termination of the ELIXIR-EXCELERATE grant.

### ELIXIR Training Portal TeSS

The **ELIXIR Training Portal, TeSS ^5^**, is ELIXIR’s flagship training service and one of its three portals. It provides a snapshot of ELIXIR’s training landscape by making training (events, materials, *etc*.) from all ELIXIR Nodes searchable and more easily discoverable in a single, central location. Significant effort has been put into providing information in ways that support user decisions and choices, allowing organisation of materials and events into training packages (groups of resources that address a particular training topic), and training workflows (navigational tools that visualise learning steps and link to resources specific to the training tasks). Work is ongoing to develop links with ELIXIR’s e-learning resources. To date, TeSS includes more than 6,000 training resources (including upcoming and past training events, and training materials) automatically aggregated from 30 content providers, including ELIXIR Nodes and third-party organisations, such as SWC/DC, GOBLET, Coursera, FutureLearn, *etc*.; TeSS content also feeds into other dissemination services, such as iAnn widgets ^6^ and Biocider ^7^. Training resources within TeSS are linked to content from other ELIXIR registries, such as tools in the bio.tools registry ^8^, and databases, standards and policies from Biosharing ^9^. TeSS is jointly developed by the University of Manchester (Prof Goble and Prof Attwood) and the University of Oxford’s e-Research Centre (Dr Sansone). TeSS is aligning its efforts with comparable initiatives worldwide (see the “External liaisons” section), and the TeSS platform is to be adopted by EMBL-Australia.

***Future of TeSS.*** Work on TeSS was initiated in the BBSRC Training award, and continues in the ELIXIR-EXCELERATE project. TeSS is a Node Service provided by ELIXIR-UK to ELIXIR, and will be subject to a Service Delivery Plan (in preparation) to a Service Delivery Plan. It is one of the three major, flagship Portals of ELIXIR. After 2019, the ELIXIR-EXCELERATE award will conclude; however, the TeSS will still need to be supported. The expectation of ELIXIR is that this is an obligation to be shouldered by the UK Node, as other National Nodes support key resources, but national funding has yet to be secured.

### ELIXIR Training Platform leadership and coordination

ELIXIR-UK has played an ELIXIR-wide strategic role in training by co-leading and coordinating, together with ELIXIR-NL (Dr van Gelder) and ELIXIR-CH (Dr Palagi), the ELIXIR Training Platform [Prof Ponting (Lead) and Dr Hendricusdottir (Coordinator), from 2014 to 2017; and currently Dr Bellis (Coordinator)]. Dr Rustici is currently contributing to the Training Platform leadership until ELIXIR establishes a procedure for the election of Platform leaders and new leaders are elected.

ELIXIR-UK’s leading role in this Platform has had an impact on the UK training strategy as well as the ELIXIR wide strategy, and has helped shaping the ELIXIR training program, establishing its aims and priorities, and developing activities/resources that address the training need of the ELIXIR scientific community.

Also, ELIXIR-UK (Dr Larcombe and Dr Hendricusdottir), supported by the Swiss (Dr Palagi) and Dutch (Dr van Gelder) Nodes, established the ELIXIR Training Coordinator Group (TrCG), representing 15 ELIXIR Nodes, which grew to 21 members representing all of the ELIXIR nodes in 2017. Dr Hendricusdottir was elected Chair of the TrCG and coordinated/co-led this group until 2017. The ELIXIR TrCG was established to coordinate training efforts across ELIXIR, sharing best practices in training and representing the interests of national nodes in pan-European activities. The TrCG made an important contribution to the H2020 ELIXIR-EXCELERATE grant proposal in which Dr Hendricusdottir coordinated the training work package (WP11). The TrCG is now an integral part of the ELIXIR Training Platform.

In addition, ELIXIR-UK (Dr Hendricusdottir) co-wrote the ‘ Coordinator guideline’ and ensured that the TrCG worked alongside the ELIXIR Technical Coordinator Group (TeCG) and had as much weight in the governance as the TeCG. Dr Hendricusdottir also contributed to and co-wrote many ELIXIR documents, including annual reports, the industry strategy, the Training Platform Road map and the ELIXIR website.

## External liaisons

Forming close ties with third-party organisations is an important outreach activity for ELIXIR as a whole, as it helps to bring wider perspectives and to avoid costly duplication of effort. This has been particularly important for the Training Platform, which has developed joint training strategies with several organisations, including (i) GOBLET, a foundation dedicated to providing a global, sustainable support and networking infrastructure for bioinformatics trainers and trainees, and (ii) the NIH-funded Big Data to Knowledge (BD2K) Training Coordinating Centre (TCC). **Both of these agreements were initiated and coordinated by ELIXIR-UK** (Dr Hendricusdottir). ELIXIR-UK has also pump-primed the Bioschemas initiative for training material specifications (in collaboration with the Pistoia Alliance), which has now developed into a flagship project for the ELIXIR Interoperability, Data and Tools platforms, and spawned an international community activity under the W3C.

### GOBLET

The **GOBLET-ELIXIR joint training strategy** sets out four key areas for collaboration:

1. work together on ‘train-the-trainer’ and ‘train-the-researcher’ activities;
2. jointly explore training 'accreditation' mechanisms;
3. share best practices and expertise on professionalising bioinformatics training; and
4. form close links between ELIXIR's TeSS and GOBLET's training portal ^10^.

In particular, **GOBLET and ELIXIR-UK have been collaborating on developing best practices and guidelines to enable training material sharing, dissemination and reuse**. As mentioned above, common standards for describing training materials have been proposed and applied to the curation of existing HTS training materials during two face-to-face meetings of trainers from the HTS and metagenomics communities. The first workshop, sponsored by ELIXIR-UK, resulted in the creation of a Git repository for sharing annotated materials, which can now be reused, modified or incorporated into new courses ^4^. All curated materials are discoverable through the TeSS and GOBLET portals. This work has helped shape the Bioschemas specifications for training materials.

The establishment and adoption of best practices for training materials is of fundamental importance, as it ensures that materials are properly described and easily comparable, for the benefit of users. Therefore, ELIXIR-UK will continue to collaboratively refine standards for training material dissemination, and apply them to a growing body of materials, starting with the EXCELERATE/GOBLET/GTN hackathon on Galaxy training material re-use that took place at the University of Cambridge in May 2017. This work will ultimately be beneficial to TeSS, enhancing material browsability and discoverability.

### Bioschemas

Bioschemas aims to leverage off-the-shelf approaches to structured Web mark-up so that search engines and metadata harvesters can extract metadata that can easily be published by providers.

Bioschemas defines sets of properties for life-science training entities, such as training materials and events, and data entities, such as data repositories, data-sets, beacons (infrastructure to allow genomic data centres to make data discoverable), samples, phenotypes and protein annotations. The Bioschemas project is a high-profile activity for the ELIXIR Interoperability platform, with major input from ELIXIR-UK institutions, including the University of Manchester (Prof Goble), the University of Oxford (Dr Sansone), Heriot-Watt University (Dr Gray) and the Earlham Institute (Mr Horro and Miss Artaza). The work crosses over to the Data and Tools platform, and is part of a wider initiative drawing in major search-engine projects ( *e.g*., Google) and the NIH BD2K BioCADDIE Centre.

Bioschemas was thought up by ELIXIR-UK and ELIXIR-Hub, and launched at the ELIXIR-UK 2015 All-Hands meeting, to overcome difficulties in feeding metadata from third-party training resources into TeSS.

### NIH BD2K Training Coordination Centre

In collaboration with the BD2K TCC, the Training Platform has developed the **ELIXIR Training Platform and BD2K TCC Training Collaboration**, which outlines five areas for collaboration between these two initiatives:

1. synergistic development of training portals: ELIXIR’s TeSS and BD2K’s BigDataU;
2. development of core competencies in bioinformatics;
3. organisation of data science summer schools in collaboration with RDA-CODATA;
4. collaboration with GOBLET; and
5. fostering international interactions and frameworks for developing standards for curating and disseminating educational materials.

The interaction of ELIXIR, GOBLET and BD2K TCC will be of direct benefit to the overall data science communities, as well as investigators seeking a broad basis for training in large-scale biomedicine around the world.

Future collaborations between the ELIXIR Training Platform and BD2K TCC were discussed in the upcoming “International Workshop on Data Science Training: Standards, Schemas, and Successes”, May 24–26, which brought together training experts from Europe, South Africa and Australia to join forces in key areas involving (i) training metadata standards, (ii) sharing content, resources and tools, (iii) training workflows, and (iv) sharing software, APIs and technical know-how/expertise.

## Additional funds

### ELIXIR-EXCELERATE

ELIXIR-EXCELERATE is a four-year project (due to end in 2019) that was awarded to accelerate the implementation, and support early operation, of the ELIXIR research infrastructure (see the first periodic report for more in-depth information). The Training Work Package (WP11) is co-led by ELIXIR-CH, ELIXIR-NL and ELIXIR-UK (Prof Ponting - from 2014 to 2017 - and currently Dr Rustici). As already mentioned, several ELIXIR-UK training resources and initiatives are dependent on ELIXIR-EXCELERATE funding, including Bioschemas, TeSS and the impact/quality assessment work. It is currently unclear what funding will be available after 2019 to continue supporting these activities and ensure their sustainability.

## Future plans

**The training landscape in the UK has changed significantly since ELIXIR and ELIXIR-UK began their activities**. We now have a wide network of universities, training centres and training initiatives across the UK, that deliver impactful training programmes, addressing the ever-changing training needs of the UK scientific community. Three of these centres have been named ELIXIR-UK Node resources, and contribute to the ELIXIR-wide training programme. Running these programmes requires training providers to be rooted in the scientific community, and to run training-gap analyses on a regular basis to ensure that training is developed and delivered in a timely way. **Training centres need to work together and in a coordinated fashion** to ensure the provision of a national training programme that is coherent and comprehensive. In many cases, the **demand for** a particular type of **training** (SWC, DC and analysis of HTS data, to name just a few) **greatly exceeds the capability of a single training centre to meet such demand**, both in terms of personnel and financial resources. Although this type of training is recognised as fundamental, **funding to support training activities is extremely limited, often sacrificed to support research activities, and training centres have to operate under cost-recovery models**. The situation is exacerbated by the fact that demand is growing, and more training providers – universities in particular – are turning to bioinformatics training centres to fill the bioinformatics training gaps in their undergraduate- and graduate-training programmes ^11^. Coordination at a national level is therefore of paramount importance to ensure that demand is met, offer is diversified, and the necessary resources needed to sustain bioinformatics training at the national level are identified and financially secured.

The ELIXIR-UK Node is currently seeking funding to hire a new Training Coordinator, to continue providing both training coordination at the national level and training outreach to external training initiatives. Appropriate funding is required not only for coordinating training activities across the UK, but also to (i) consolidate the work already done, (ii) enable the development of new training activities, (iii) support existing training centres and their initiatives, (iv) maintain our international commitments to support the ELIXIR Training Portal, and (v) participate in an influential way in the ELIXIR Training Platform.

Specifically, appropriate funding (supporting key personnel) needs to be secured to sustain the following fundamentally important activities:

1. Sustain the development and maintenance of TeSS: as previously indicated, this currently relies on the ELIXIR-EXCELERATE award, which terminates in 2019. National funding needs to be secured to sustain this flagship ELIXIR portal.
2. Sustain the activities of the ELIXIR-UK Training centres (Cambridge, Birmingham and Edinburgh). As presented above, these deliver crucial training in priority areas, reaching a significant number of users (>2,500) every year, but they are operating under extremely limited budgets and cost-recovery models, which are insufficient to ensure long-term sustainability. Moreover, as they are Node contributions to the ELIXIR-wide training programme, they must adhere to ELIXIR’s standards for quality, discoverability and interoperability, fulfilling their obligations to ELIXIR, without receiving any support from it. In some cases, the centres are the sole providers of training on a particular topic. For example, metabolomics training provision is extremely limited across Europe, and the Birmingham Metabolomics Training Centre has developed several, successful face-to-face courses. These have high running costs and, in order to make them affordable, NERC is funding some bursaries, allowing NERC PhD students or early-career scientists to attend a course for free, while the running costs are recovered from NERC. This model has been extremely efficient and appropriate, as the training becomes free to those in need of it, and the metabolomics community's knowledge grows. We would recommend other Research Councils to consider implementing the same approach for "their" early-career researchers. Additional funding solutions need to be identified to sustain the provision of metabolomics training.
3. Continue playing an influential role in the ELIXIR-wide training programme. ELIXIR-UK contributes key services to the ELIXIR training programme, leads the training impact assessment work, and established the ELIXIR Training Coordinator Group, to ensure the provision of a coherent bioinformatics programme across ELIXIR Nodes. The TrCG is a unique feature of the ELIXIR Training platform, providing great support in running its activities; it represents a vital forum for Training Coordinators across ELIXIR to discuss collaborations, align initiatives and leverage each other’s expertise. ELIXIR-UK needs to be represented on the TrCG, otherwise it will miss out on this crucial networking opportunity. This role is currently fulfilled by Dr Bellis and Dr Rustici (University of Cambridge), but this is not a sustainable solution.
4. Coordinate the provision of bioinformatics training across existing, and new, ELIXIR-UK training centres and initiatives. ELIXIR-UK will launch another ‘Expression of Interest’ exercise for new resources to become part of ELIXIR-UK in 2018, and we foresee that new training centres will apply to join. Consequently, the coordination effort will need to expand to include such centres, as well as training initiatives like the BBSRC Bioinformatics and Biomathematics Training Hub, a collaborative project to coordinate the development and sharing of training materials and expertise across the UK’s National Institutes of Bioscience (NIB), which is now seeking partnership with ELIXIR-UK to sustain its activities and make them discoverable through TeSS.
5. Ensure that appropriate training solutions (including training materials and face-to-face training events) are put in place for showcasing ELIXIR-UK resources. With the recent increase in the number of recognised ELIXIR-UK resources (including data, tools and interoperability resources), we want to ensure that these are included and represented in training activities. In some cases, this might involve facilitating activities in which ELIXIR-UK resources are represented; in other cases, it could mean providing guidance on developing new training solutions.
6. Facilitate and support the development of training materials/solutions in areas where ELIXIR-UK has strong expertise, including but not limited to:
  • *Data management and stewardship*: this has been identified as a high priority area by the ELIXIR Training Platform, and significant expertise is available in the UK Node in this domain: *e.g.*, FAIRDOM (led by Prof Goble), which supports ISA-based project data and model management, and is partnering with ELIXIR-NO, ELIXIR-DE and ELIXIR-NL. A proposal to develop collaborative training solutions in this area has been drafted between the Oxford e-Research Centre (BioSharing, Dr Sansone), TeSS and the University of Cambridge (Dr Rustici), but no funds are currently available to enable this;
  • *Structural bioinformatics training workflows*: structural bioinformatics expertise in the UK Node is significant, and the desire to contribute to training activities is high; so far, however, this has not been possible owing to lack of funds and dedicated resources. Collaborative work is planned to implement structural bioinformatics workflows in TeSS (with Prof Orengo, UCL), but additional funding is needed to consolidate these activities. These are just one example of training workflows that could be implemented in TeSS; TeSS is actively pursuing the inclusion of a growing number of workflows, and the means to link them to external resources.
7. Run SWC/DC courses at an increasing number of UK sites, to satisfy a growing demand for this basic training. The SSI’s role in establishing the SWC/DC ELIXIR training programme has been pivotal, but the SSI is currently struggling to continue providing the coordination needed at the national level, given the extreme popularity of this training and the growing demand. Resources are needed to ensure that SWC/DC training is delivered to the UK scientists who need it, to support the organisation of courses, and increase the pool of UK trainers that can deliver this type of training, thereby increasing capacity. In the long term, this approach might enable the inclusion of such core training in a growing number of teaching/training programmes.
8. Actively engage the UK scientific community. Resources are needed to engage with the UK community at large, to ensure that training activities are tailored to its needs. Regular gap analysis must be carried out to understand the training gaps across the rapidly evolving research landscape. This is a vital process, to ensure that training programmes are kept up to date. Community-engagement activities should target a diverse range of stakeholders, including academic and industry partners, both as consumers of and contributors to training activities. The ELIXIR Industry Advisory Committee has highlighted industry engagement as a priority for the coming year. To get these activities kick-started, ELIXIR-UK will host an SME event in January 2018 (Cambridge, UK), and will sponsor the UK Bioinformatics Core Facilities meeting, a nascent initiative that is trying to coordinate the activities of core facilities across the UK, and with which ELIXIR-UK wishes to engage.
9. Continue leading the development of training best practices, to increase training quality, and help users to discover training solutions that best meet their needs. As the number and type of training resources increase, so does the degree of difficulty in finding the training that is most appropriate for trainees’ skill levels and expectations. Mapping existing training to bioinformatics core competencies would not only help trainees to choose the training that best fits their needs, but would also provide clear learning paths, signposting what training activities they should attend over a certain period of time to acquire the competencies needed to carry out their work effectively. The ELIXIR Industry Advisory Committee (IAC) has identified this as an area that should be developed in TeSS. This exercise would also contribute to refining existing core competencies (ISCB) ^12^ and expand them as needed. ELIXIR-UK is currently in the process of proposing an Implementation Study focusing on developing solutions to signposting training through core competencies and learning paths.
Additionally, ELIXIR–UK needs to play a leading role in the development of national, coherent training curricula in priority areas such as clinical bioinformatics, data management and stewardship, *etc*. In doing so, it should establish strategic partnerships with relevant initiatives, such as the MRC Health Data Research UK for clinical bioinformatics.

## Conclusions

Since 2014, a significant amount of work has been done within ELIXIR-UK to coordinate, strengthen and expand bioinformatics training across the UK and ELIXIR as a whole. Crucial partnerships and collaborations have been established with several external initiatives, fostering interactions with international partners, and aligning efforts on a global scale.

Training remains a high priority for ELIXIR-UK and significant effort will be put into securing funding and recruiting a new Training Coordinator to drive training activities, to initiate new ones, and to continue participating in an influential way in the ELIXIR Training Platform.

## Supplementary material

Supplementary File 1: Table with original timetable of activities from the BB/L005069/1 grant.
