# Supplementary figures and images for: ELIXIR-UK role in bioinformatics training at the national level and across ELIXIR
Source: F1000Res. 2017 Jun 21;6:ELIXIR-952. [Version 1] doi: 10.12688/f1000research.11837.1 (PMC5521157; doi:10.12688/f1000research.11837.1)

**Supplementary File 1: Table with original timetable of activities from the BB/L005069/1 grant.**
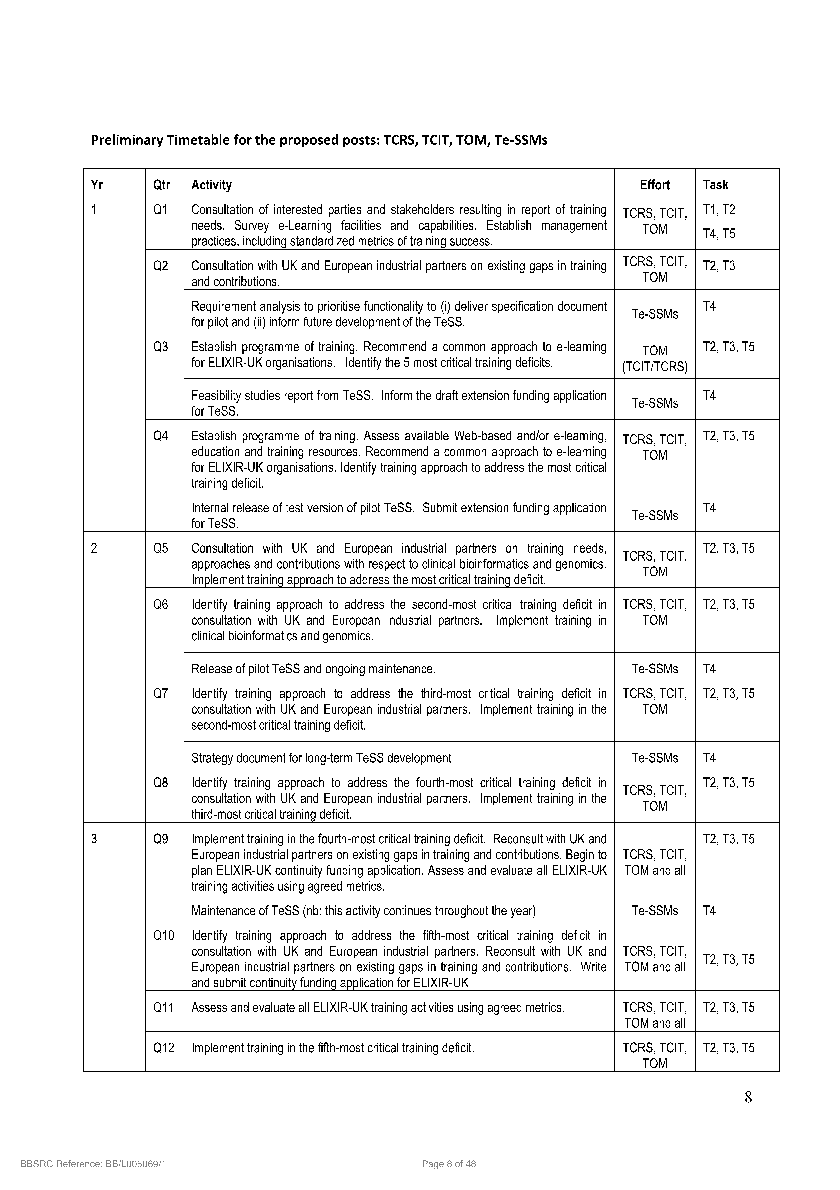

Supplement: Supplementary file 1 [file f1000research-6-12791-s0000.tgz › 469ed880-1a69-4304-88ca-87523f085726.docx]
