# Supplementary material for: ELIXIR-UK role in bioinformatics training at the national level and across ELIXIR
Source: F1000Res. 2017 Jun 21;6:ELIXIR-952. [Version 1] doi: 10.12688/f1000research.11837.1 (PMC5521157; doi:10.12688/f1000research.11837.1)
Supplement: Peer review history

# Peer review: Referee response for version 1

**DOI:** 10.5256/f1000research.12791.r23887

**Published:** 2017-08-14

**Authors:** Manuel Corpas

**Affiliations:** 1 Repositive, Cambridge, UK

The article "ELIXIR-UK role in bioinformatics training at the national level and across ELIXIR" reflects the developing training opportunities that ELIXIR-UK, as part of its training mandate, is performing throughout the UK and ELIXIR-Europe. My inside knowledge as Technical Coordinator for ELIXIR-UK from 2014-1016 may have influenced my review of this paper.

 My main comments:

- I concur with another reviewer comments that the paper lacks a coherent structure. I do not see a clearly stated ELIXIR-UK’s training strategy and how it is being coherently being fleshed out throughout the article. Instead, the reader is presented with a chronological description of activities according to the different grants that support the training work of the node. Different sections would benefit from more cohesion between them with some kind of 'story' thread that joins them. Thus I am not sure if this is a summary technical report or a review of the training activities for the node. 
- ELIXIR-UK has more activities than training and it would add clarity to the narrative if the other activities in the node are enumerated in addition to how ELIXIR-UK’s training leadership has helped in developing them. 

A few explicit remarks now follow:

- You say “ELIXIR-UK is at the forefront of bioinformatics training provision, across the UK and in the wider ELIXIR training programme” (Page 3, 1st paragraph) but you do not qualify it. Is there any explicit independent confirmation of this?
- What is the ELIXIR-UK training strategy? Explicitly, what does it consist of? What is the plan, achievements, goals and milestones? How do you know you are being successful?
- How is the ELIXIR-UK Training operations building the community of bioinformatics in the UK? You mention this as one of its key priorities but miss the detail on how this is happening. This is related to my previous comment on the strategy development and is alluded to briefly in the community engagement paragraph of future work (page 9, bullet point 8) but I fail to clearly grasp current efforts.
- You mention the Training programs in Cambridge, Birmingham and Edinburgh. They seem disconnected. What are your plans to synchronise these resources at a national level other than just being named services?
- Even though the complete skill surveys are referenced, it would add credibility to the summary statistics of the survey if its design and people involved both in developing and responding it are also included.
- You say that you support the interoperability platform of ELIXIR, which is an external activity to training. What is the take of ELIXIR-UK training strategy to abide by FAIR-ness of data and materials involved in these courses/workshops? In what way are training courses/committed committed to FAIR values?
- Since FAIR is a cross-cutting initiative throughout ELIXIR and has so many ramifications in the spread of best practices, I feel this crucial aspect is missing in the paper (see above).
- Can you substantiate/measure in what way the excellent feedback from SWC/DC workshops has been collected and summarised?
- Page 7 paragraph 2: “co-wrote many ELIXIR documents, including annual reports, the industry strategy, the Training Platform Road map and the ELIXIR website” - I would appreciate references for this.

Thanks for giving me the opportunity to review this paper. The amount of activity presented here is indicative of the many efforts carried out by all ELIXIR-UK partners. By giving this paper more shape and a story thread, I am convinced it will provide an authoritative account of your contributions to training in the UK, Europe and beyond.

I have read this submission. I believe that I have an appropriate level of expertise to confirm that it is of an acceptable scientific standard.

# Peer review: Referee response for version 1

**DOI:** 10.5256/f1000research.12791.r23885

**Published:** 2017-07-27

**Authors:** Nicola J. Mulder

**Affiliations:** 1 Computational Biology Group, Department of Integrative Biomedical Sciences, Institute of Infectious Disease and Molecular Medicine, University of Cape Town, Cape Town, South Africa

This paper provides a comprehensive description of the ELIXIR-UK training activities and plans, though it was unclear whether the aim was to focus on ELIXIR-UK or ELIXIR overall, as the focus changed between these in the paper. It describes a well thought out process for evaluating training needs and attempting to address these. Challenges and future plans are discussed, and the issue of funding is raised repeatedly. The paper is interesting and useful, though I have a few minor concerns raised below.

 The paper is quite long and I think some fine details could be removed, as the reader would not necessarily be interested in every name of who did what. The continual reference to funding issues is a bit annoying for the reader, these could be discussed once off in a section under challenges.

 On page 6 there is mention that it is unclear how impact assessment would be supported after the grant ends, but if there is funding for training then it is not much extra overhead to assess the courses. Personal interviews would not be necessary for every course if resources are limited.

 Page 7 - mention of working with BD2K on developing core competencies, is the group also working with the ISCB core competency group? Is the BD2K portal not called ERuDIte now, rather than BigDataU?

 Page 8 line 2: "...ND2K TCC were discussed in the upcoming.." doesn't make sense. The meeting has now already taken place. Note, the USA was of course also involved among the countries listed.

 For long term sustainability and addressing the demands, there could perhaps be a stronger focus on training trainers, and potentially live streaming some courses to cover a wider audience.

I have read this submission. I believe that I have an appropriate level of expertise to confirm that it is of an acceptable scientific standard.

# Peer review: Referee response for version 1

**DOI:** 10.5256/f1000research.12791.r23698

**Published:** 2017-07-19

**Authors:** Celia van Gelder

**Affiliations:** 1 Dutch Techcentre for Life Sciences (DTL), Utrecht, Netherlands

This paper gives a good overview of the various and important contributions of ELIXIR-UK both to training in the UK node and to the ELIXIR Training Platform as a whole. It also gives insight in the coordination efforts that are needed throughout the UK to achieve a coherent training infrastructure. Throughout the paper it touches several times upon a major challenge that everyone in training and training coordination is facing, which is the struggle to get sustainable funding in place to ensure continuity and sustainability for the training activities. It will be a waste of time and effort if the training infrastructure that has been built will simply stop after specific projects end. It would be interesting to learn a bit more about the ambition of and opportunities for ELIXIR-UK to achieve a national approach to this sustainability challenge. Is the ELIXIR-UK structure helping to achieve this goal?

 The ELIXIR Training Platform activities are built by coordinating efforts in all the ELIXIR nodes and as such is a combined effort of many countries. It would be good to make this a bit clearer to the reader, e.g. in the section where the ELIXIR Training Platform is introduced. In addition a reference to https://zenodo.org/record/61411 could be included, which gives detailed background information about the setup of the ELIXIR Training Platform.

 Here are some of my specific comments and suggestions:

  

- There is a mentioning of three ELIXIR flagship portals, but it is not explained which three portals the authors are referring to. This might confuse the reader.
- Related to the TeSS portal it could be useful to give some insight in visitor statistics and also make the distinction between events and materials more clear, as well as the distinction between future and past events and materials. It should be realized that a large number of the 6000 entries mentioned are related to events that happened in the past. As such TeSS represents an important training archive in bioinformatics, and to my knowledge this is the only portal that offers this kind of information.
- Related to the ELIXIR-SWC/DC agreement:
   
  - Lesson development will definitively take place and will be taken up by the ELIXIR nodes, but this not an explicit part of the partnership agreement that is currently being prepared for signing.
  - The agreement contains workshops and instructor trainings. The agreement entails 2 workshops per ELIXIR node, and not 2 workshops per year per ELIXIR node, as is currently stated.
  - The authors could refer to the F1000 paper about the ELIXIR SWC/DC pilot that has now been published. ^1^
- Related to establishing the GOBLET-ELIXIR collaboration agreement it would be good to mention that Teresa Attwood, who is both Chair of GOBLET, and part of ELIXIR-UK, played a major role.
- Related to the description of the process during WP11 grant writing it would be good to stress that this was a collaborative process of the WP11 leaders and several node representatives.
- In the section on BioSchemas the training implications could have been described clearer. Further, this section could refer to the FAIR (Findable, Accessible, Interoperable, Reusable) principles, where BioSchemas mainly addresses findability.

I have read this submission. I believe that I have an appropriate level of expertise to confirm that it is of an acceptable scientific standard.

## References

[1] PawlikAvan GelderCNenadicAPalagiPKorpelainenELijnzaadPMarekDSansoneSHancockJGobleC: Developing a strategy for computational lab skills training through Software and Data Carpentry: Experiences from the ELIXIR Pilot action. *F1000Research*.2017;6: 10.12688/f1000research.11718.1 10.12688/f1000research.11718.1 28781745PMC5516217

# Peer review: Referee response for version 1

**DOI:** 10.5256/f1000research.12791.r23696

**Published:** 2017-07-17

**Authors:** Patricia M. Palagi

**Affiliations:** 1 SIB Training Group, Swiss Institute of Bioinformatics (SIB), Lausanne, Switzerland

This article describes an extensive and comprehensive report of the ELIXIR-UK training resources and the ELIXIR-UK role in defining ELIXIR’s training. It is very useful to other ELIXIR nodes and other countries willing to setup a bioinformatics training program. The manuscript is clear and well written, but it is often read as a long administrative report for funders. Hereafter you will find my suggestions, mainly to reduce redundancy and to improve clarity:

 General remarks:

- Long term sustainability for training is a major issue, in UK and in several countries, and it is continuously repeated in this report. Instead of repeating at each session, it could have a much higher impact if it was stated only once, in the future plans only, with a full paragraph/session dedicated only for this.
- It would be useful to give more details on the funding opportunities the authors are envisioning and exploring to sustain training in the long term.
- Besides from funding, what are the other issues that ELIXIR-UK are facing regarding training? For instance, number of well-trained trainers and for each areas of expertise?
- The commitment from the expert areas is only explicitly stated in the Applied Genomics session. Is community commitment an issue in the other areas of training?

In the five thematic areas:

- The training content in the five thematic areas are of direct interest to other countries and could be better detailed in their respective sessions.
- In the session Statistics training, what is taught in the statistics skill schools? Does it go beyond what is covered in the STATO didactic tool?

 

 Supplementary material:

 The table in the supplementary materials is difficult to understand and does not support the content. There are acronyms that are not explained (TCRS, TOM, etc) and the time span is not anchored in the actual calendar. It would be worth: adding actual time periods, explaining the acronyms (or removing them if they are not useful for the content), explaining the tasks or removing them. This lighter and more self-explanatory version of the table (which could become an image) could then support the content of the manuscript and be inserted within the text, as a summary.

 Some minor corrections:

- Some texts are highlighted in bold ( **ELIXIR-UK has initiated and coordinated the provision of SWC/DC training across ELIXIR**; etc), but it seems to be an error (or at least the reason why is not explicit).
- In the Future of TeSS, “to a Service Delivery Plan” is written twice.
- In “Future collaborations between the ELIXIR Training Platform and BD2K TCC were discussed in the upcoming “International Workshop on Data Science Training: Standards, Schemas, and Successes”, May 24–26, “, the word upcoming is outdated. 

I have read this submission. I believe that I have an appropriate level of expertise to confirm that it is of an acceptable scientific standard.

# Peer review: Referee response for version 1

**DOI:** 10.5256/f1000research.12791.r23886

**Published:** 2017-07-14

**Authors:** Michelle D Brazas

**Affiliations:** 1 Bioiniformatics, OICR, Toronto, Ontario, M5G 0A3, Canada

This article provides an update on the ELIXIR-UK role in setting up and providing training resources and tools for both the UK and the broader ELIXIR community. It provides a good overview of the activities conducted and work achieved since 2014. The article then also provides an opinion of the funding situation supporting training activities.

 Some general comments for improvement:

1. The title and abstract indicate that the article will be about ELIXIR-UK and its role as lead of ELIXIR Training, and the work it has accomplished both for the UK and ELIXIR in this training sphere. The introduction even ends with an excellent summary statement "This report provides an overview of ELIXIR-UK's role in bioinformatics training at the national level." However, the article scope goes beyond what is indicated by the abstract, and frequently includes funding issues and statements. While I recognize the need to acknowledge funding sources (can be done under Grant Information section) and to highlight funding shortfalls, I am not convinced that the current organization of repeating funding sources and problems in each section is the ideal organization as it appears to be a complaint rather than a constructive opinion. Grouping all funding related content into one section (Future Plans section) would greatly improve the read and tone of the article. In fact, splitting the article in 2 would be ideal - one article would be the report, as described by the abstract; and the second article would be the opinion, and could describe the future directions/funding opinions expressed at the end of the current article and throughout each section.
2. Some references are hyperlinks and others are superscripts to the Reference Section. It would be better to be consistent. For example, the GOBLET survey on page 3 is a hyperlink to the publication rather than being listed in the reference section.
3. Some resources are missing hyperlinks, particularly the ELIXIR-UK website on page 3. Please add hyperlinks to text missing them. Another example on page 6, for core Key Performance Indicators identified by the group. A hyperlink (or further text on the KPIs) to the KPIs in particular would be helpful to groups looking to implement similar metrics in their own training programs.
4. What is the model for resources used in the statistics training and metabolomics training? Is the training software free or commercial software? How does commercial software align with the mandate of ELIXIR-UK and ELIXIR to train broadly?
5. Page 5, Clinical Genomics, are there not other institutes like EBI involved in Clinical Bioinformatics training? As a North American, EBI comes to mind as an important training centre in the UK, yet is not discussed in this article. In general, where do these other very visible training groups in the UK fit into ELIXIR-UK?
6. Page 7, External Liaisons - keep the same order of relationships as presented in the opening paragraph for this section. GOBLET section - the establishment and adoption of best practices is fundamental to what or whom? Bioschemas section - awkward location of the last paragraph. Consider moving up within the section. NIH section - North America was also a training expert brought together here. Please add.
7. Page 8, Future Plans - how is it known that the delivered training programs are impactful? What are the metrics of impactfulness? Is this related to the KPIs? If so, please hyperlink or share the KPIs so that other training groups may benefit from these. Why is the cost-recovery model not sustainable? The point of a cost-recovery model is to ensure sustainability, otherwise the model is wrong.
8. Page 8-9, Future Plans - in general the future plans section focuses more on the UK activities than the ELIXIR wide activities. Only the ELIXIR-wide TeSS and Coordination activities are described as going forward. Is the training impact and quality assessment work not continuing, and why not?

Overall, this article describes a significant body of work and expresses important opinions on funding its continuation, but would greatly benefit from a reorganization, and possibly a split into two articles - report and opinion.

I have read this submission. I believe that I have an appropriate level of expertise to confirm that it is of an acceptable scientific standard, however I have significant reservations, as outlined above.
